# S4-3 Protocol for assessing implementation of physical activity policies by national governments using the Physical Activity Environment Policy Index (PA-EPI)

**DOI:** 10.1093/eurpub/ckad133.020

**Published:** 2023-09-11

**Authors:** Kevin Volf, Catherine B Woods

**Affiliations:** Physical Activity for Health Research Cluster, Health Research Institute, University of Limerick, Limerick, Ireland; Department of Physical Education and Sport Sciences, University of Limerick, Limerick, Ireland

## Abstract

**Purpose:**

The implementation of physical activity policies has been slow and uneven. As a remedy, the World Health Organization has recommended that national policy implementation should be supported with practical tools and guidance. The Physical Activity Environment Policy Index (PA-EPI) is a tool that allows the extent of policy implementation to be assessed. It comprises a list of good practice policy indicators. These are categorized into eight policy domains targeting aspects of the physical activity environment and seven infrastructure support domains for strengthening physical activity policy systems. This protocol provides researchers with guidance on how to use this tool.

**Project:**

Guided by a protocol developed by the International Network for Food and Obesity / Non-communicable Diseases (NCDs) Research, Monitoring and Action Support (INFORMAS), the PA-EPI protocol. The INFORMAS protocol was modified based on the research teams experience carrying out the first study utilising the PA-EPI tool.

A multi-step process is recommended. This process relies upon the identification of two groups of partners: national policymakers and independent stakeholders tasked with the promotion of physical activity directly and/or indirectly. During the early steps of the process the desk-based research is undertaken to identify information about and evidence of physical activity policy implementation- the PA-EPI Evidence Document. This mapped information is presented to the national policymakers to verify that it is valid and comprehensive. Subsequently, the evidence of implementation is presented to the stakeholders who, using a 5-point Likert scale (1-little or none to 5 high), individually rate the extent of policy implementation in practice. These individual ratings are aggregated and a implementation scorecard is generated displaying the extent of implementation of each of the good practice indicators Stakeholder then engage in an expert workshop to review implementation scores, and identify key policy implementation recommendations which are presented to government.

**Conclusions:**

The process of conducting a policy implementation assessment using the PA-EPI requires the cooperation of several stakeholders as part of a national coalition. It is envisioned that PA-EPI assessments will enable international cross comparison. This will promote learning from international best practice.

